# Generalized Parking Occupancy Analysis Based on Dilated Convolutional Neural Network †

**DOI:** 10.3390/s19020277

**Published:** 2019-01-11

**Authors:** Sherzod Nurullayev, Sang-Woong Lee

**Affiliations:** Pattern Recognition and Machine Learning Lab., Gachon University, 1342 Seongnamdaero, Sujeong-gu, Seongnam 13120, Gyeonggi-do, Korea; sfreedom9393@gmail.com

**Keywords:** parking occupancy detection, dilated convolution, PKLot, AlexNet, CarNet

## Abstract

The importance of vacant parking space detection systems is increasing dramatically as the avoidance of traffic congestion and the time-consuming process of searching an empty parking space is a crucial problem for drivers in urban centers. However, the existing parking space occupancy detection systems are either hardware expensive or not well-generalized for varying images captured from different camera views. As a solution, we take advantage of an affordable visual detection method that is made possible by the fact that camera monitoring is already available in the majority of parking areas. However, the current problem is a challenging vision task because of outdoor lighting variation, perspective distortion, occlusions, different camera viewpoints, and the changes due to the various seasons of the year. To overcome these obstacles, we propose an approach based on Dilated Convolutional Neural Network specifically designed for detecting parking space occupancy in a parking lot, given only an image of a single parking spot as input. To evaluate our method and allow its comparison with previous strategies, we trained and tested it on well-known publicly available datasets, PKLot and CNRPark + EXT. In these datasets, the parking lot images are already labeled, and therefore, we did not need to label them manually. The proposed method shows more reliability than prior works especially when we test it on a completely different subset of images. Considering that in previous studies the performance of the methods was compared with well-known architecture—AlexNet, which shows a highly promising achievement, we also assessed our model in comparison with AlexNet. Our investigations showed that, in comparison with previous approaches, for the task of classifying given parking spaces as vacant or occupied, the proposed approach is more robust, stable, and well-generalized for unseen images captured from completely different camera viewpoints, which has strong indications that it would generalize effectively to other parking lots.

## 1. Introduction

The noteworthy growth of the automotive industry together with a lack of urban planning have caused problems such as traffic congestion, air contamination, and driving difficulties. To facilitate consumers, most car manufacturers provide a pre-installed or aftermarket navigation system in vehicles. This enables drivers to easily navigate to their destination. However, navigation systems provide a rough estimate of the remaining distance to the destination.

With the recent technological advancements in sensing and intelligent technologies, companies are interested to find out whether these advancements can assist in reducing the time spent to search for vacant parking spaces. Locating an unoccupied parking space is a major predicament that drivers face since searching it is a tedious process. Thus, systems that can detect vacant parking spaces will enable drivers to efficiently locate vacant parking spaces. Such systems are referred to as, parking guidance systems (PGSs). PGS can decrease the time consumed by drivers in searching for a parking space and help them avoid driving impediments. As a subsidiary effect PGSs relieve overall traffic congestion in city centers, consequently, decrease fuel consumption and CO emissions. The goal of PGS is to obtain the most credible and definitive data from parking vacancies and provide them in a form that is most helpful for consumers. PGSs differ considerably in their detection performance, optimization, and reporting process. Our goal is to improve the detection performance of a PGS. Three principal factors should be considered when selecting a detection scheme. Robustness is the primary factor. A robust indicator should accurately provide the status of a parking space, despite the diversity in environmental parameters, such as temperature, and the varieties of cars and parking slots. Second, maintenance is an additional determinant that must be considered, together with the cost of new tools to be purchased per parking stall, the deployment effort, and the necessity to restrict normal parking throughout the installation period. Third, the number of sensing units clearly has a direct effect on the establishment and maintenance expenses of a detection system.

To resolve this problem, researchers have proposed methods can be characterized as counter-, sensor-, and image-based. Counter-based methods compute the number of cars entering and exiting the parking section. Thus, systems based on these methods include gate-arm counters and inductive loop detectors positioned at the gates of parking facilities. The systems’ display panels show reports and directions and guide the driver toward a vacant parking space. This type of arrangement can notify the total number of unoccupied spaces; however, it does not guide a driver to the exact location of a vacant parking space. An alternative approach is to use sensors (such as ultrasonic, inductive loop, and infrared laser sensors), which can be pre-installed in each parking space for indicating its occupancy status. These sensors are normally reliable; however, the principal disadvantage of sensor-based methods is the cost of building the system because a great number of sensor units are needed to cover the entire parking area. A vision-based detector can overcome the shortcomings of sensor per-stall and counter-based detection methods. A vision-based system is an optimal choice since its installation is manageable and it demands no closedowns. Moreover, computer vision for traffic applications is an emerging area of research, including notable advances in the last few years. For instance, intelligent transportation management systems, like those investigated in [1], attempt to achieve tasks such as tracking, detection, and identification of vehicles, as well as higher-level assignments like comprehending vehicle behaviors and exposing irregularities. In a vision-based system, every visual node which includes a camera and a transmitter can observe multiple vehicles concurrently, decreasing the cost per parking stall. As it can be adopted for other tasks, such as surveillance, the basis of the system is usually already installed, and it can be managed by other applications after deployment. However, those who advocate sensor-based methods, such as the authors of [2], claim that video cameras are considerably more expensive than sensors and produce a large volume of data that may not be easily disseminated through a wireless network. On the contrary, a review of the literature, [3], revealed that image-based parking reservation detection policies can be deployed using extant surveillance cameras that are already attached to a primary monitoring system. Thus, it appears that image-based systems are a valid choice, especially for very large or outdoor parking areas where the installation of hundreds of sensors is impracticable. In consideration of this, many vision-based parking space management systems have been discussed and developed recently. Frequently, researchers turn to solve the issue with deep learning since it can extract features and learn to classify automatically. Deep networks, such as convolutional neural networks (CNNs), are robust to lighting variations and diverse circumstances. Nevertheless, without adjustment, a standard CNN cannot operate for all possible conditions. Thus, to create a robust parking space location system, we should take many difficulties into account, such as lighting variations, varying vehicle sizes, illuminance, perspective distortion, inter-object occlusion, camera resolution, and unfavorable weather. Most previous efforts were focused on resolving these difficulties. However, this task cannot be simply generalized and indeed the adaptation of a specific solution to an unknown parking lot is not easy.

In this paper, to address the aforementioned issues, we introduce a CNN structure that uses dilated convolutional neural networks to indicate parking space occupancy status. The PKLot dataset [4], which is publicly accessible, was used to evaluate the performance of our model. The use of images of different scenarios and parking areas allowed us to test the generalizability of our approach. We trained our model on one subset of images and tested it on a completely different one. Several approaches already exist, such as that investigated in the study reported in [5], mAlexNet, where this type of generalization property was tested experimentally; however, we show that our method outperforms them. Most of the related studies compared their approach with AlexNet [6], which has demonstrated a high-level performance on this task. Thus, we analyzed our approach in comparison with AlexNet, as well as with other well-known deep learning structures.

The remainder of the paper is organized as follows. Section 2 reviews the related work. The features of the dataset we used in our experiments are described in Section 3. In Section 4, we provide a detailed explanation of our classification method. Our experiments and the obtained results are described in Section 5. Finally, Section 6 concludes the paper.

## 2. Related Work

**Figure 1:**
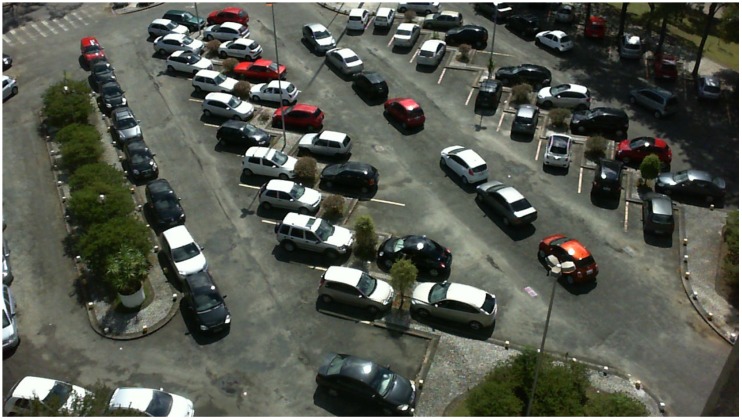
Example for a car parking area taken from PKLot dataset [4].

To overcome the problem described above, there currently exist various systems, such as those proposed in [2,7,8,9,10,11,12,13,14,15,16,17,18,19,20,21,22,23]. In some studies, the parking management system problem was addressed by using smart devices [24,25,26,27]. Sensor-based systems involve detection sensors, such as ultrasonic sensors, which are installed at each parking space [11,28,29,30]. Huang and Wang [31], stated that image-based methods can be categorized into two classes: space-driven and car-driven. Space-driven methods are focused on identifying vacant parking places rather than cars [8,32]. For images obtained from static (such as the surveillance) cameras, the strategy most frequently applied is background subtraction [33], which assumes that the variation in the background is statistically motionless within a small interval of time. Considering this assumption does not hold for outdoor scenes, the limits of this approach are immediately clear. A more robust method was proposed in [34]. The authors used Gabor filters to train a classifier with images of unoccupied parking spaces under various lighting conditions. In the car-driven method category, algorithms are trained to recognize automobiles, which are the objects of interest. Related to this issue are a few object detection methods that were proposed in [35,36]. Because of the perspective distortion observed in most images of parking lots, such as that shown in Figure 1, cars located at a considerable distance from the camera occupy a small area in an image, and thus, fewer details can be observed, which considerably diminishes the performance of object detection algorithms. The method introduced in [5], is based on a deep Convolutional Neural Network specifically designed for smart cameras. They achieved fairly high results and we will be comparing our results with respect to it. The method, called mAlexNet, is a version of AlexNet and offers a decentralized solution for visual parking space occupancy detection. It achieved very good results, but only for the same subset of images. In [37], the problem of occlusion of parking spaces, where one or more parking spaces can be hidden by other parked vehicles, was addressed. To solve the occlusion problem, vehicle tracking to detect the events of a car entering or exiting a parking space has been suggested. Furthermore, in addition to methods that use visual techniques and ground sensors, methods exist that use sensors installed on cars or carried by drivers. For instance, in [38], a framework for detecting parking space occupancy in which the system collaborated with in-vehicle navigation systems was proposed. In [39], a crowd-sourcing solution, leveraging sensors in smartphones, was proposed for collecting real-time parking availability data. In the context of vehicle detection, the single method of which we have knowledge that uses a CNN is that presented in [40]. It uses a multi-scale CNN to detect vehicles in high-resolution satellite images. In addition to providing a dataset, the authors of [4], addressed the problem by applying machine learning techniques. They used their dataset, which contains approximately 700,000 images of parking spaces in parking lots captured by three different cameras, to train SVM classifiers on various textural features, such as LBP, LPQ, and their variations. They additionally enhanced the detection performance by using composites of SVMs, applying simple aggregation functions, such as maximum or average, to the confidence values given by the classifiers. We will be comparing our work with respect to it. In the method presented in [41], specifically trained customized neural networks were used to ascertain parking space occupancy statuses and parking demand based on visual features extracted from images of parking spaces. The method’s performance evaluation, conducted in a 24-h period and based on 126 parking spaces, showed that the classifier yielded promising results for night and day. The authors of [42], used a Bayesian hierarchical structure to formulate a vacant parking space detection scheme, which is based on a 3D model for parking spaces, that can operate day and night. Likewise, the design presented in [43], models every parking space as a volume in 3D space and is thus able to consider occlusions when estimating the probability that a vehicle is present in a parking space. In [44], the authors attempted to remove the occlusion problem from which the former approach suffers by classifying the state of three bordering spaces as a unit and using a color histogram of the three spaces as the feature for their SVM classifier. To handle the problem of lighting changes, in the study [45], a Bayesian classifier based on corners, edges, and wavelet features was applied to verify the detection of vehicles. Moreover, in [46], methods that use aerial images for detecting vacant parking spaces were presented. The authors proposed a self-supervised learning algorithm that automatically obtains a set of canonical parking space templates to learn the appearance of a parking space and estimates its structure from the learned model. In [47], LUV color space, gradient magnitude and six quantized gradient channels are used as features and feed into machine learning methods, Linear Regression and SVM to detect the status of a given parking place. Since the results they achieved are high, we will be comparing our work with respect to it. The limitation of this method is its relatively low detection accuracy.

## 3. Datasets

### 3.1. PKLot Dataset

By reason of the vast number of possible applications, parking lot status detection schemes have attracted a great deal of research attention in the last decade. However, in the computer vision field, the lack of datasets that can be used to assess and analyze proposed algorithms is a problem that researchers face frequently. An example of a parking area is shown in Figure 1.

**Figure 2:**
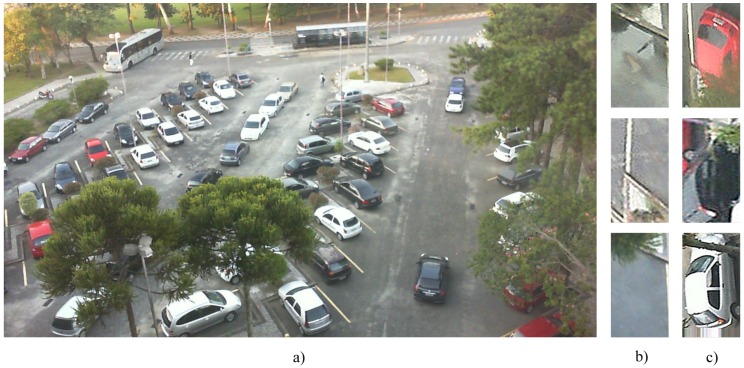
(**a**) example of a parking lot in PKLot dataset [4]; (**b**) example images of empty parking spaces after cropping and rotating; (**c**) example images of occupied parking spaces after cropping and rotating.

To overcome this difficulty, as mentioned above the contributors of [4], introduced the PKLot dataset, which offers an important resource for researchers and practitioners whose objective is to create outdoor parking space vacancy detection systems. As our goal is to classify parking space occupancy, the PKLot dataset is one of the most appropriate candidate resources for facilitating this task, providing images captured in two parking lots from three separate camera views. It succeeds in solving the problem regarding the lack of a common dataset, allowing future benchmarking and evaluation. This dataset contains images captured on sunny, rainy, and overcast days, and thus, we found it a very useful tool for our research purpose. The PKLot dataset contains 12,417 images of parking areas and 695,899 images of parking spaces segmented from them, which have been manually checked and labeled. All the images were captured in the parking areas of the Federal University of Parana (UFPR) and the Pontifical Catholic University of Parana (PUCPR), both located in Curitiba, Brazil. The cameras were located on the roof of the buildings to decrease the potential occlusion of adjacent cars. Moreover, such settings allow series of pictures that vary widely in terms of changing illumination caused by weather variations to be obtained. To build a more challenging dataset, in [4], images presenting difficulties, such as images of vehicles that are overexposed because of sunny conditions, images with shadows caused by trees, and images collected during heavy rain that resemble pictures captured at night because of the lack of natural light, were included in the dataset. An essential detail of this dataset is that only legal parking spaces were marked and segmented. The parking lots those are signed (delimited) with parallel yellow or white lines on the floor are considered as legal ones. As one may remark, there might be some automobiles parked in an illegal manner like in the middle of the street. The pictures were saved in JPEG color format with lossless compression in a resolution of 1280 × 720 pixels. The pictures taken from the cameras were cropped by the authors of [4], into single parking space images, rotated and grouped. Some of the examples of occupied and vacant parking spaces are shown in Figure 2.

**Figure 3:**
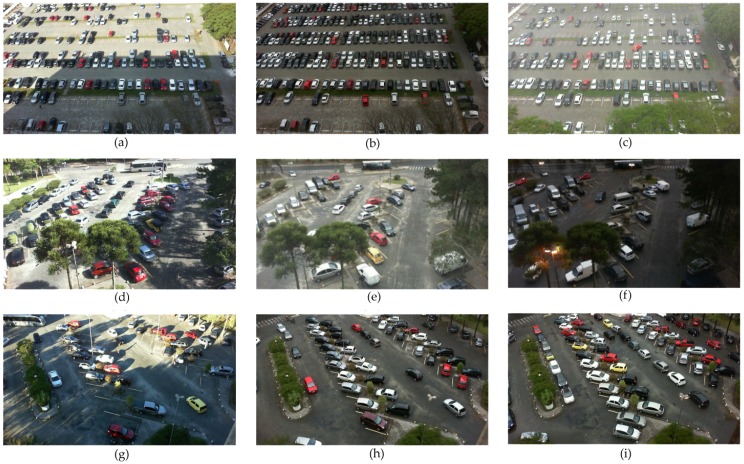
Examples of photos taken under various weather conditions [4]: (**a**–**c**) from PUCPR (sunny, overcast, rainy respectively); (**d**–**f**) from UFPR04 (sunny, overcast, rainy respectively); (**g**–**i**) from UFPR05 (sunny, overcast, rainy respectively).

All the cropped images were arranged into three subsets by the authors of [4] and named as UFPR04, UFPR05, and PUCPR. The first two subsets contain pictures of varied views of the same parking area captured from cameras located on the 4th and 5th floors of the UFPR building. Images captured from the 10th floor of the administration building of PUCPR constitute the third subset. Our investigations showed that UFPR04 is a more challenging subset than UFPR05 and PUCPR because it contains images with diverse obstacles and ground patterns. The subset contains 3791 images captured under the aforementioned weather conditions. To these images, a semi-automatic segmentation process was applied and they were also manually labeled. A total of 105,845 images of individual parking spaces, 43.48% occupied and 56.42% vacant, was generated during the process. A very similar report was produced for the PUCPR and UFPR05 subsets. In [4], all three subsets, PUCPR, UFPR04, and UFPR05, were categorized manually into overcast, sunny, or rainy subfolders according to the observed weather conditions. Figure 3 shows some example images of the three parking areas obtained under the before-mentioned weather conditions.

The available images show a wide luminance variation because they were captured under different climatic conditions. They were used for examining the performance of classifiers trained on images of a single parking lot. A brief review of the factors that distinguish this dataset and render it useful is as follows:

- The captured images show various parking areas having dissimilar features.
- Generally, commercial surveillance system cameras are placed at a high above the cars, and therefore, they can observe all the vehicles in a certain parking place. However, this makes our task more challenging, and therefore, the cameras were positioned at various heights to obtain a variety of images.
- The images were captured under diverse weather conditions, such as sunny, rainy, and overcast, which represent various illumination conditions.
- Various types of challenges, such as over-exposure due to sunlight, differences in perspective, the presence of shadows, and reduced light on rainy days, are provided by the current dataset images.

### 3.2. CNRPark + EXT Dataset

The dataset provides a great facility to researches containing nearly 150,000 labeled pictures of empty and used parking spaces. A smaller dataset of approximately 12.000 designated images, CNRPark [21], is covered and noticeably enlarged by CNRPark + EXT, reference [5]. Reference [21] holds parking lot pictures obtained in various days of July 2015, from 2 different cameras A and B, which were installed in order to have diverse viewpoints.

**Figure 4:**
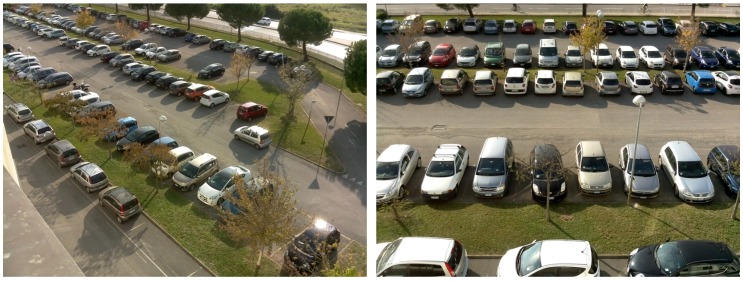
Example photos from CNRPark + EXT dataset [5].

Similarly, CNRPark dataset is also publicly accessible. The CNRPark + EXT is a significant expansion of CNRPark dataset with images obtained from November 2015 to February 2016 under different weather circumstances by 9 cameras with distinct angles of view. Examples are given by Figure 4.

**Figure 5:**
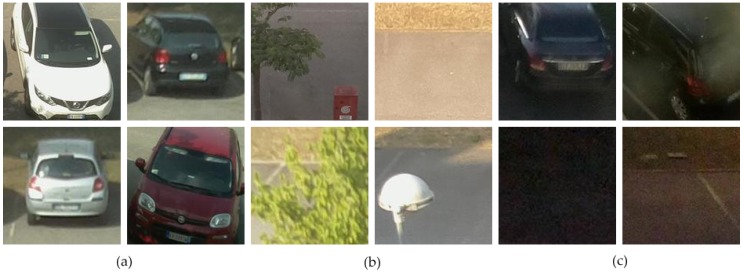
Parking spaces (patches) from CNRPark + EXT dataset [5]: (**a**) occupied patches; (**b**) empty patches; (**c**) dark or night time patches.

Partial occlusion patterns due to obstructions such as lampposts, trees, other cars, and different lighting conditions are involved in this dataset. See Figure 5. This enables training a classifier that is able to characterize most of the challenging circumstances that can be detected in a real-life scenario. Similar like PKLot dataset, CNRPark dataset also provides cropped images from pictures taken by cameras making our work easier. Each image represents a single vacant or occupied parking space. Examples of these patches are given in Figure 5.

The parking patches might be relatively farther or nearer depending on the distance from the camera. All of them are in a square size. The authors of the dataset manually labeled all the parking spaces according to the status of the occupancy. Similar to PKLot dataset, patches of the CNRPark + EXT dataset are arranged into subsets according to various weather circumstances such as sunny, rainy and overcast. Furthermore, training, validation and test subsets are provided for classification. However, we slightly modified these folders on the coding process. In the CNRPark + EXT dataset, 4287 camera images were obtained in 23 diverse days, and these pictures are cropped into 144,965 labeled parking space patches. Table 1 reports an information about the number of patches in CNRPark + EXT and PKLot datasets.

In PKLot, images are derived applying rotated rectangular masks. Unlike it, in CNRPark + EXT dataset, parking spaces are not rotated and mostly do not include parking space fully or accurately. Moreover, CNRPark + EXT contains heavily occluded spaces such as almost completely hidden by trees or lampposts. However, these obstacles are less influential in the set of segmented spaces of PKLot dataset. Besides, pictures were captured from a lower point of views with respect to PKLot, providing extra occlusions due to next automobiles. One of the very important features of CNRPark + EXT dataset is that it contains relatively darker and night time images as well. It helps to make training images diverse and create environment similar as in real-life scenarios. See last four image patches in Figure 5.

## 4. Proposed Architecture

### 4.1. Brief Overview of AlexNet Structure

To detect efficiently, we propose CarNet, which uses dilated convolutional layers and we examine its representation with respect to the AlexNet [6]. It is designed into five convolutional layers, some of which are followed by max-pooling layers and two fully connected layers with a final 1000-way softmax. The simplification of the network is also justified by the fact that the original AlexNet architecture was composed for visual recognition tasks which are more difficult than our binary classification problem. Freshly, AlexNet was trained on the 1.2 million high-resolution images in the ImageNet LSVRC-2010 dataset to classify them into the 1000 different classes. Although the network shows a high-level performance on the current task, we consider that its structure is too deep for classifying only two groups of elements and, thus, a more robust structure can be realized by focusing primarily on the extracting high-frequency features. In our situation, we must distinguish only two classes: whether or not a vehicle exists in a given parking space image. In fact, the results reported in this paper prove that our proposed design can easily and effectively handle the car parking occupancy detection problem.

### 4.2. Dilated Convolution

**Figure 6:**
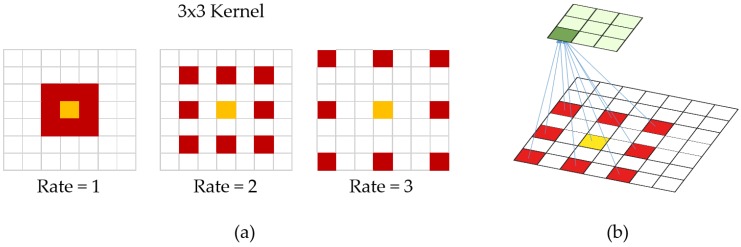
(**a**) examples of dilated convolutions. When dilation rate is 1, it becomes normal convolution. (**b**) 3D appearance of dilated convolution (rate = 2).

Let’s assume we are given very blurred patch of a parking space. Despite our eyes cannot detect a car in the patch, we can say that there must be car depending on high frequency patterns. However, we might not see car in the image. Reversely, if there is no car in the image it should be somehow smooth and hold less frequent features. If parking space image holds less frequency patterns, we may predict that there is not car. However, vanilla (normal) convolutions struggle to integrate global context. A recent development [48], introduced one more hyperparameter to the convolution layer called the dilation. In simple terms, dilated convolution is just a convolution applied to input with defined gaps. With this definition, given input is an 2D image, dilation rate k = 1 is normal convolution and k = 2 means skipping one pixel per input and k = 3 means skipping 2 pixels. See Figure 6. Dilated convolution is a way of increasing receptive view (global view) of the network exponentially and linear parameter accretion.

With this purpose, it finds usage in applications cares more about integrating knowledge of the wider contextual information with less cost. Furthermore, a model uses dilated convolution runs faster than a model uses normal convolution. These are where the idea of using dilated convolutions came from to solve the given issue. As an example, a very good proposal presented by [49], which is applied for action segmentation and detection. Dilated convolution is applied in domains beside vision as well. One good example is [50], text-to-speech solution and [51] learn time text translation. They both use dilated convolution in order to capture global view of the input with less parameters. Ref. [50] applies dilated causal convolutions to raw audio waveform for generating speech, music and even recognize speech from raw audio waveform.

### 4.3. CarNet Structure

**Figure 7:**
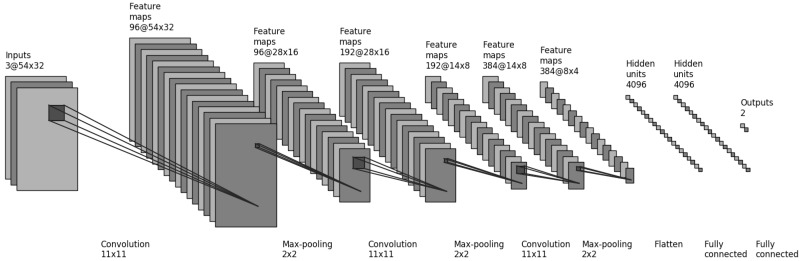
CarNet structure.

The name CarNet inspired by our goal—to detect car occupancy in a parking lot. A more detailed description of the structure of CarNet is shown in Figure 7. The contributions of the proposed study are three-fold.

1. We use dilated convolutional layers to build our architecture. A brief overview of dilated convolution is provided in Figure 6. The reason we use dilated convolution is we have to avoid learning too deep. Dilated convolution allows a model to learn large features while ignoring small ones. It resembles filtering mostly high-frequency features.
2. We use large kernel sizes to learn features from parking lot images. As we know from deep learning that larger windows sizes cause larger features to be learned. In our experiments, we therefore applied a large window size. Comprehensive details are provided in the following sections.
3. We investigated empirically the optimal number of layers that is perfectly suited for solving the current issue. A small number of layers, three, is used in our CarNet. If it contains too many layers, a model learns too deeply. Our task is not to classify 1000 groups, as is that of AlexNet, but is simpler because we have only two classes. Thus, learning too deep would lead to a failure. We proved this in our experiments, as described in Section 5. Moreover, most previous deep learning approaches, including [5], used a small number of layers, mostly three.

In each of the convolutional layers used by CarNet, large windows sizes, 11 × 11, are used, and dilated convolution is applied; the dilation rate is 2. We implement three fully connected layers including the output layer. The number of units in the first and second fully connected layers is 4096. The final one, the output layer consists of 2 units because our task is binary classification. All three convolutional layers are supported by linear rectification (ReLU) and max-pooling process. CarNet shows a trend that the number of filters increases layer by layer. We propose using 96 filters in the first dilated convolutional layer. The second layer consists of 192 filters, which is a dramatic increase. Finally, the last layer’s depth is 384. It is noticeable that every next layer is doubled compared to the previous one. Dropout regularization is used before each fully connected layer, including the output layer. We used strong dropout regularizations in the fully connected layers to avoid overfitting. This renders CarNet generalizable and robust for various subsets of images. Whereas in AlexNet local response normalization is used, in our method we do not apply any type of normalization. Our model is not as deep as AlexNet and the images we use for training are fairly small and thus we might lose some important features if we applied normalization methods. This omission is required for building a model that is robust to camera view images that differ from the training images.

As input, the proposed architecture takes a 54 × 32 RGB image, and therefore, before training, parking space images may need to be cropped and resized. The reason is that the dimension of all the parking space images in PKLot is small because small parking space images were extracted from the large images captured by the cameras. The minimum height and width are 58 and 31 pixels, respectively, while maximum height and width are 176 and 85 pixels, respectively.

Moreover, it takes extremely less time to train CarNet, due to the facts that the input size of images is very small, the window sizes of the convolutional network are considerably large and small number of layers. However, strong hardware support is not needed to train our model. The training time for 500 epochs is around five hours. For the training our model, we used an open source neural network, Keras. GeForce GTX 1080 Ti graphics card was used in our experiments on CarNet. More detailed information about the parameters we used in our experiments is as follows. The batch size is 64 and the number of epochs is 500. To optimize our training, we use Stochastic Gradient Descent algorithm and parameters are: learning rate is 0.00001, weight decay is 0.0005, momentum is 0.99. Furthermore, when we experience our model, we trained a certain subset of the PKLot dataset and tested on different one, to show the effectiveness of our model. This makes our approach special. Because CarNet demonstrates high performance on an unseen subset of images. Moreover, when we train our model, we use five-fold cross-validation. We divided a certain subset of the dataset into five parts and 80% was used for training while 20% for validation. In addition, shuffling was performed after each epoch.

## 5. Experimental Results

### 5.1. Exploring an Optimal Architecture—CarNet, by Making Experiments on PKLot Dataset

**Figure 8:**
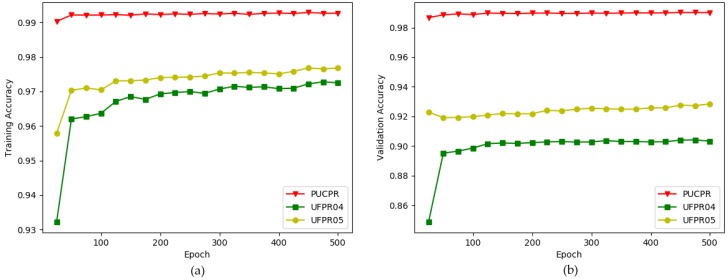
Comparison of subsets of PKLot dataset [4]. x-direction: number of epochs (500), y-direction: accuracy. (**a**) training accuracy; (**b**) validation accuracy.

To explore a model which fits to the current problem, we made experiences on PKLot dataset and later evaluated this model with another dataset, CNRPark + EXT. Initially, we compared the subsets of the PKLot dataset to analyze them and confirm the challenging nature of each. The indications for training accuracy and validation accuracy are given in Figure 8. The first impression is that the best performance is for PUCPR, showing the highest achievements in training and validation accuracy. The scores of UFPR04 and UFPR05 are clearly lower. The exact scores are provided in Table 2.

However, the performances in the testing phase show that among the subsets UFPR04 contains the most challenging images, which contain more obstacles, shadows, trees, etc. An additional proof of this is that training with PUCPR, UFPR04, and UFPR05 and testing on UFPR04 shows the lowest accuracy results, 94.4%, 95.6%, and 95.2%, respectively. See Table 2.

**Figure 9:**
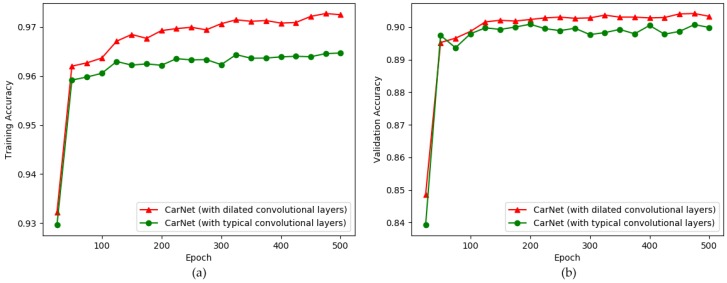
Comparison of CarNet (with dilated convolutional layers) and CarNet (with normal convolutional layers). x-direction: number of epochs (500), y-direction: accuracy. (**a**) training accuracy; (**b**) validation accuracy.

**Figure 10:**
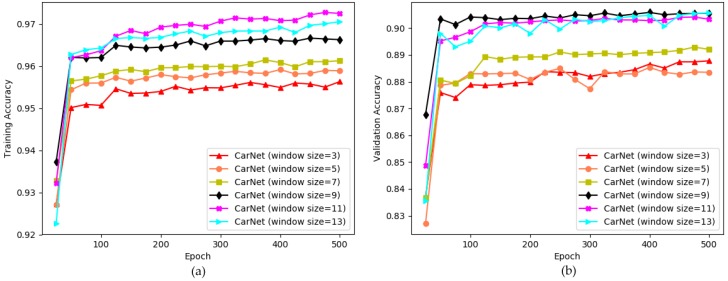
Experimenting different window sizes to build an optimal model. x-direction: number of epochs (500), y-direction: accuracy. (**a**) training accuracy; (**b**) validation accuracy.

**Figure 11:**
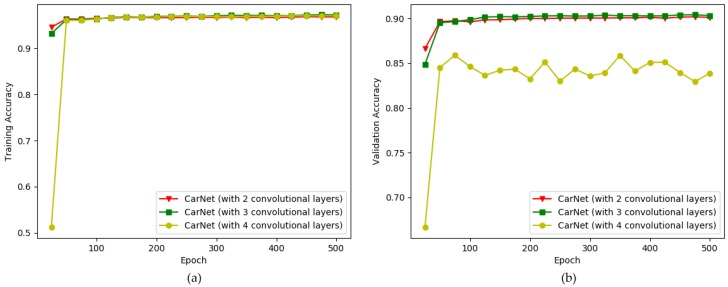
Comparison of CarNet (with two layers), CarNet (with three layers) and CarNet (with four layers). x-direction: number of epochs (500), y-direction: accuracy. (**a**) training accuracy; (**b**) validation accuracy.

**Figure 12:**
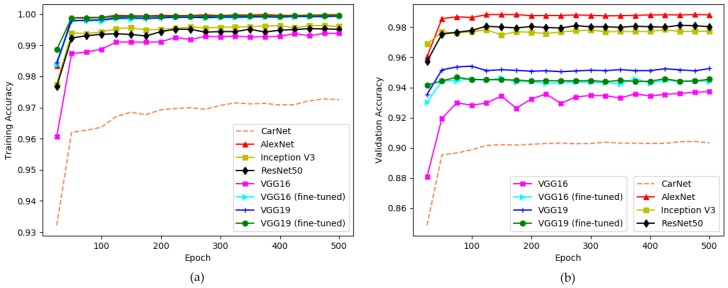
Experiment on PKLot [4] dataset. Comparison of our model with respect to other models. x-direction: number of epochs (500), y-direction: accuracy. (**a)** training accuracy; (**b**) validation accuracy.

When we trained CarNet with all the subsets and tested them on PUCPR, the highest scores were achieved, which means that it is the least challenging subset. For this reason, the training scores for PUCPR are the highest. It became clear that UFPR05 is the second most challenging subset. It is obvious that the more challenging images we use, the more robust model we can build. Nevertheless, when we trained our method with PUCPR and tested it with two different subsets we achieved very promising results. As UFPR04 is the most challenging subset, we conducted our experiments using this subset. Overall, we conducted five types of experiments including Figure 8. The reader may ask why we used 500 training epochs in our experiments. The answer is that using this number of epochs was beneficial to able to differentiate the performances of the models clearly, as well as to obtain better results. In some diagrams, the differences between the lines cannot easily be distinguished and some lines even exchange their position with other lines. For instance, the performance scores are not clear at the finishing point of 100 epochs in the graphs in Figure 9a,b as well as Figure 10a,b. Furthermore, even sharp changes can be observed in the graphs in Figure 11a,b as well as in Figure 12a,b.

As the use of dilated convolution is one of the main contributions of our study, we conducted an experiment to demonstrate the impact of using dilated convolutional layers rather than using layers without dilated convolution. See Figure 9. In this experiment, we trained CarNet (with dilated convolutional layers) and CarNet (without dilated convolutional layers). Instead of dilated convolutional layers, we used normal convolutional layers; the other properties were the same as those of CarNet. Dilated convolutional layers, as previously mentioned, help to avoid the situation where the model learns very small features by focusing on more general (high-frequency) features. Until epoch 40 there was no difference between the models with dilation and without a dilation.

In Figure 9a, it can be observed that after the final epoch the training and validation accuracies for CarNet (with convolutional layers) were 97.23% and 90.70%, respectively, whereas the results for CarNet (without dilated convolutional layers) show training and validation accuracies of 96.47% and 89.99%, respectively. See Table 3.

As shown in the line graphs in Figure 9, initially the model without dilated convolutional layers shows a rapid increase in accuracy. However, in the final epochs the trend for CarNet (with dilated convolutional layers) shows a slight improvement over CarNet (without dilated convolutional layers. Table 4 shows the testing accuracies related to Figure 9.

The table indicates that CarNet is dominant in all cases, showing the highest scores. A noteworthy fact is that results for CarNet (without a dilation) shows a quite good result. This explains the importance of applying the two other contributions of this study, that is, a large window size and a small number of layers, in that they have a significant influence when the current issue is addressed. Thus, the model shows high performance even when dilated convolutions are not used.

Furthermore, the use of a small number of layers is an additional main contribution of our study, as mentioned previously. Thus, we conducted an experiment on models with varying numbers of convolutional layers to find the model that optimally fits the current task. Figure 11 illustrates a comparison of the scores for the training and validation sessions of CarNet (with two convolutional layers), CarNet (with three convolutional layers) and CarNet (with four convolutional layers). At a glance, one cannot easily determine which method shows the highest performance, especially when comparing CarNet (with two convolutional layers) and CarNet (with four convolutional layers). As seen in Figure 11, the line for CarNet (with three convolutional layers) shows a comparable best performance in (a) graph, demonstrating the highest training accuracy as well as the highest validation accuracy in (b) graph.

With very small difference, the line for CarNet (with two convolutional layers) have the second highest scores. This proves that using less layers might be helpful for this task. Meanwhile, the lowest performance belongs to CarNet (with four convolutional layers. The training and validation scores are respectively 96.82% and 90.12% for CarNet (with two convolutional layers), while the scores for CarNet (with four convolution layers) are respectively 97.06% and 83.88%. See Table 5. Our proposed method contains three convolutional layers, for which we demonstrated the training and validation accuracy above.

In Table 6, the testing scores for CarNet (with two convolutional layers), CarNet (with three convolutional layers), and CarNet (with four convolutional layers) are provided to demonstrate the effect of number of layers. The scores for CarNet (with two convolutional layers) and CarNet (with three convolutional layers) are higher than those for CarNet (with four convolutional layers). This shows that the use of fewer layers is more suited to our task. However, we determined that the optimal number of convolutional layers is three rather than two because the model with three convolutional layers yielded higher scores.

We previously claimed that the use of a large window size in convolutional layers helps to solve our classification task efficiently. However, we do not know exactly how large should kernel size be. We proved that the kernel size that we chose is the best option for solving the current challenge. The comparison results are shown in Figure 10.

The figure indicates that we selected the appropriate window size for solving the current problem. The exact training and validation accuracies for this experiment is provided by Table 7.

We started our experiments training with a model which uses 3 × 3 kernel size and continued until experimenting a model which uses 13 × 13 kernel size. The lines representing the accuracy for window sizes 3 and 5 show the lowest training accuracy as well as the lowest validation scores. Meanwhile, the charts for the performance of CarNet (window size = 7) indicate average performances compared to all the other results, in both diagrams, Figure 10a,b. The scores for models which use 9, 11, 13 window sizes are comparable the highest ones in each diagram showing that using larger window sizes is more effective.

The testing scores are given in Table 8. The testing results for CarNet (with window size = 3), CarNet (with window size = 5), CarNet (with window size = 7), CarNet (with window size = 9), CarNet (with window size = 11), CarNet (with window size = 13) are provided by the table. It is difficult to differentiate the performances clearly. However, overall it can be seen that the accuracies for models with larger window sizes indicate that these models are more robust. However, we found that an 11 × 11 kernel size is the most appropriate option for the parking space occupancy detection problem.

### 5.2. Comparison of CarNet with Respect to Well-Known Architectures by Making Experiments on PKLot and CNRPark + EXT Datasets

Last but the most important type of experiments we made are to compare the results of our network with those of other well-known architectures, including AlexNet, which showed a noteworthy performance on the current task. To assess proposed architecture, we evaluated our model with two datasets, PKLot and CNRPark + EXT. However, we only used PKLot dataset to build most reliable model. In this section, experiments on CNRPark + EXT are also provided to consider how well generalized our model is.

#### 5.2.1. Experimental Results on PKLot Dataset

Training and validation accuracies on PKLot dataset are provided by Figure 12.

As can be seen in the figure, in all three-line graphs deeper models show higher scores. The exact accuracy values are provided in Table 9. The lines for VGG16 and VGG19 describe the training accuracies of these models trained from the scratch while the patterns for VGG16 (fine-tuned) and VGG19 (fine-tuned) illustrate the scores of these models, which we fine-tuned pre-trained weights trained on ImageNet. A few conclusions can be obtained from the results shown in Table 9. The highest scores for both training and validation accuracy were achieved by AlexNet, 99.98% and 98.82% respectively, while the scores for CarNet are the lowest, 97.23% and 90.70%. However, AlexNet is not the deepest model, but rather has a smaller number of layers than the other models in the table. This proves that deeper models do not yield better solutions on this task and inspired our idea to use fewer layers.

Thus, rather than conducting experiments on deeper networks, we decided to develop a model with fewer layers. Furthermore, that a model shows the lowest training and validation accuracy does not mean that it is the least effective model. The graphs indicate only the training phase accuracy. The performance shown by the other methods may also mean that they are overfitting. The experimental results shown in Figure 12, and prove that CarNet demonstrates a higher accuracy than all the other models.

Finally, Table 10 shows the testing accuracies of nine experiments related to Figure 12. In the table, testing scores of the models AlexNet, CarNet, VGG16, VGG16 (fine-tuned), VGG19, VGG19 (fine-tuned), Xception, Inception V3, ResNet50 are given. An examination of these architectures in the table reveals that deep networks are very effective for solving this task. As mentioned previously, in previous studies the performances of the methods were compared with that of AlexNet. However, the results of our experiments indicate that, among these networks, except CarNet, Inception V3 and ResNet50 are the most robust. See the scores for Inception V3 and ResNet50 in Table 10. The reason why AlexNet was chosen as a comparative method is that it shows the best performance for images from the same dataset. We give examples to support this consideration and show performances in training/testing format from the scores for AlexNet in Table 9. The score for PUCPR/PUCPR (training/testing) is 98.6%, which is the second highest after that of CarNet. Next, the score for UFPR04/UFPR04 (training/testing) is 98.2%, which is the highest among all the architectures we used in our experiments. The score for UFPR05/UFPR05 (training/testing) is 98%, which is also higher than that for all the other networks. However, AlexNet shows poorer results when we trained with one subset of images and tested with a different subset. For instance, the score for UFPR04/PUCPR is 89.5% and the score for PUCPR/UFPR05 is 83.4%, as shown in the table. Although in two out of the nine cases AlexNet’s performance is better than that of other models, our proposed method, CarNet, shows more robustness, demonstrating the best results in seven out of the nine cases. See the testing results for AlexNet and CarNet in Table 10.

#### 5.2.2. Experimental Results on CNRPark + EXT Dataset

**Figure 13:**
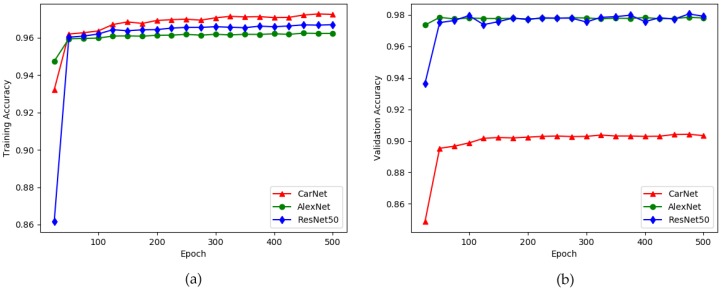
Experimenting on CNRPark + EXT dataset [5] to build an optimal model. x-direction: number of epochs (500), y-direction: accuracy. (**a**) training accuracy; (**b**) validation accuracy.

We trained Carnet, AlexNet and ResNet50 to evaluate how good is our model. Since these are the models which demonstrate highest testing scores on PKLot dataset we assume that making experiments only with these models would be enough. Training and validation accuracies of these architectures are given in Figure 13.

The exact scores related to Figure 13 are provided by Table 11. Similar like training on PKLot dataset, the table also show that AlexNet illustrates the highest result in training and validation phases. Training and validation accuracies for CarNet are 97.91% and 90.05% respectively, showing the lowest scores. The highest score is depicted by AlexNet, 96.99% and 97.91%, for training and validation respectively. The last model which showed fairly high testing accuracies on PKLot dataset, ResNet50 showed training accuracy 96.51% while showing 97.80% validation accuracy. However these are only training and validation accuracies which are not most important number to evaluate a certain model. Ultimate assessment, testing accuracy scores are given in Table 12. CarNet has the highest scores on testing phase.

Results are as we expected. Alexnet demonstrated second highest testing accuracy, 96.54%, while ResNet50 has the lowest testing score among other two architectures, with score 96.24%. The most noticeable consideration from experiments on the given datasets, deep models represent poorer results than models with less layers.

### 5.3. Comparison of CarNet with Previous Approaches by Making Experiments on PKLot and CNRPark + EXT Datasets

We compared testing scores of CarNet with prior works mentioned in [4,5,47]. However, ref. [5] uses both datasets, PKLot and CNRPark + EXT, to experiment their proposed architecture, while [4,47] made experiments only on PKLot dataset. Moreover, ref. [5] provided their final results as a testing accuracy scores while [4,47] provided as AUC (Area Under the Curve) scores. Thus, we calculated AUC scores for CarNet experiments in order to be able to compare its performance with [4,47].

Initially, we show testing scores for CarNet and mAlexNet [5] only on PKLot dataset. Comparison results with mAlexNet [5] on PKLot dataset are given by Table 13. As one may notice, there are nine combinations of training/testing subsets. In four cases out of nine mAlexNet showed better performance than Carnet, while in another five cases CarNet outperforms mAlexNet. In the order of training/testing subsets, the cases mAlexNet has higher results are UFPR04/UFPR04, UFPR05/UFPR05, PUCPR/UFPR04 and PUCPR/PUCPR with values 99.54%, 99.49%, 98.03% and 99.9% respectively. The case CarNet achieves higher testing scores are UFPR04/UFPR05, UFPR04/PUCPR, UFPR05/UFPR04, UFPR05/PUCPR and PUCPR/UFPR05 with values 97.6%, 98.3%, 95.2%, 98.4% and 97.6% respectively. An important thing to notice here is CarNet showed better generalization while mAlexNet mostly having better testing results when they train and test on same subset of images. Final column of the table shows the average testing accuracies for both architectures. Mean accuracies for mAlexNet and CarNet are 96.74% and 97.04% respectively, showing slightly higher accuracy score by CarNet.

Following, we compared CarNet with mAlexNet on CNRPark + EXT and PKLot datasets. Testing scores for both approaches are given by Table 14.

Results are as we expected. Furthermore, We tried to test both architecture in combination of both datasets. However, mAlexNet shows very poor results when training on PKLot and testing on CNRPark or in a reverse case.

Given three combination of testing scores. In all cases, CarNet shows much more robustness. As we claimed before, the main advantage of CarNet is that it is well-generalized. It shows good performance when training on a certain dataset and testing on a different dataset.

The last type of indications we will be showing are comparisons of AUC scores for CarNet with respect [4,47] on PKLot dataset. Ref. [47] is the another recent proposal achieving one of the highest performances so far, using image processing and machine learning. The authors of [47] extracted ten features from an image: three channels of LUV color space, gradient magnitude as another feature and six quantized gradient channels. Following, they fed these features into Logistic Regression (LR) and Support Vector Machine (SVM). We will be referring the approach provided by [4] as PKLot and the approach provided by [47] as Martin et al. (LR) and Martin et al. (SVM). Comparisons of four types of scores are provided by Table 15. Among the results, Martin et al. (LR) shows best performance only in one case, training/testing for UFPR04/UFPR05. PKLot, ref. [4], demonstrates best results on three cases, training/testing for UFPR04/UFPR04, UFPR05/UFPR05 and PUCPR/UFPR05. From these results, we may conclude that PKLot is very good while training and testing on same dataset. The AUC scores for CarNet outperforms other two methods in five cases out of nine. The cases Carnet shows highest scores are UFPR04/UFPR05, UFPR04/PUCPR, UFPR05/PUCPR, PUCPR/UFPR04, PUCPR/UFPR05 with values 99.35, 99.82, 97.91, 98.45 and 99.38 respectively. Overall conclusion is that CarNet is fairly robust to train in one dataset and test on another one. We believe that it can be applied various of application to overcome challenges in real-life scenario.

## 6. Conclusions

An efficient solution—CarNet, for visual detection of a parking status, was presented which uses Dilated Convolutional Neural Networks. CarNet is provided as a robust design for employees to classify images of parking spaces taken from a camera as occupied or vacant. In this proposed approach, we used three contributions: dilated convolutional, a small number of layers, large window sizes. We showed by experiments that the current task can be tackled effectively by using these contributions. To assess the performance of CarNet and to be able to compare it with respect to other architectures, we made our experiments on PKLot dataset since prior attends were evaluated on this dataset as well. PKLot contains images with high variability related to occlusions, various point of views, illumination and weather conditions such as sunny, rainy, and overcast days. This makes the dataset more compatible with a real scenario of an outdoor parking lot, and represents a good complement to other publicly available datasets, for more reliable assessments. We performed experiments to compare the performance and generalization capabilities of our approach against other state-of-the-art techniques. These experiments confirm that our proposed method outperforms AlexNet and other well-known architectures. CarNet represents higher accuracy for totally new images compared to prior approaches. The proposed approach helps drivers to find available parking spaces quickly, and thus decreases traffic load, air pollution, and possibly illegal parking. Numerous issues merit further investigation, such as accuracy improvement of classification or rather than providing the nearest accessible parking space, providing spots with more than one available parking space may prevent the parking space being unexpectedly occupied by other drivers.

## Figures and Tables

**Table 1:** Number of images in the datasets.

Datasets	Empty Spaces	Occupied Spaces	Total
PKLot [4]	337,780	358,119	695,899
CNRPark + EXT [5]	65,658	79,307	144,965

**Table 2:** Comparisons of training and validation accuracies for subsets of PKLot dataset [4].

Name of Method	Training Accuracy	Validation Accuracy
PUCPR	99.26%	99.01%
UFPR04	97.23%	90.70%
UFPR05	97.68%	92.85%

**Table 3:** Comparison of training and validation accuracies for CarNet (with dilated convolutional layers) verses CarNet (without dilated convolutional layers).

Name of Method	Training Accuracy	Validation Accuracy
CarNet (without dilated convolutional layers)	96.47%	89.99%
CarNet (with dilated convolutional layers)	**97.23%**	**90.70%**

**Table 4:** Comparison of testing accuracies for Carnet (with a dilation) versus CarNet (without a dilation).

Name of Method	Name of Training Subset	Testing Accuracy		
		PUCPR	UFPR04	UFPR05
CarNet (with a dilation)	PUCPR	**98.80%**	**94.40%**	**97.70%**
	UFPR04	**98.30%**	**95.60%**	**97.60%**
	UFPR05	**98.40%**	**95.20%**	**97.50%**
CarNet (without a dilation)	PUCPR	94.70%	94.30%	93.80%
	UFPR04	95.60%	94.50%	92.30%
	UFPR05	95.80%	94.90%	94.10%

**Table 5:** Comparisons of training and validation accuracies for CarNet (with two layers), CarNet (with three layers) and CarNet (with four layers).

Name of Method	Training Accuracy	Validation Accuracy
CarNet (with two convolutional layers)	96.82%	90.02%
CarNet (with three convolutional layers)	**97.23%**	**90.70%**
CarNet (with four convolutional layers)	97.06%	83.88%

**Table 6:** Comparison of testing accuracies for Carnet (with two layers), CarNet (with three layers) and CarNet (with four layers).

Name of Method	Name of Training Subset	Testing Accuracy		
		PUCPR	UFPR04	UFPR05
CarNet (with two layers)	PUCPR	95.60%	94.20%	92.20%
	UFPR04	96.10%	95%	90.30%
	UFPR05	95.70%	95.10%	91.70%
CarNet (with three layers)	PUCPR	**98.80%**	**94.40%**	**97.70%**
	UFPR04	**98.30%**	**95.60%**	**97.60%**
	UFPR05	**98.40%**	**95.20%**	**97.50%**
CarNet (with four layers)	PUCPR	62.80%	89.20%	68.40%
	UFPR04	57.90%	88%	65.30%
	UFPR05	63.40%	87.50%	66.60%

**Table 7:** Comparison of training and validation accuracies for experiments with different window sizes.

Name of Method	Training Accuracy	Validation Accuracy
CarNet (window size = 3)	95.63%	88.78%
CarNet (window size = 5)	95.89%	88.35%
CarNet (window size = 7)	96.13%	89.21%
CarNet (window size = 9)	96.63%	90.55%
CarNet (window size = 11)	**97.23%**	**90.70%**
CarNet (window size = 13)	97.06%	90.56%

**Table 8:** Comparison of testing accuracies for experiments with different window sizes.

Name of Method	Name of Training Subset	Testing Accuracy		
		PUCPR	UFPR04	UFPR05
CarNet (with window size 3)	PUCPR	92.60%	92.80%	95.70%
	UFPR04	92.90%	94.10%	94.50%
	UFPR05	92.10%	94.50%	95.10%
CarNet (with window size 5)	PUCPR	94.80%	93.40%	92.40%
	UFPR04	94.20%	93.60%	92.90%
	UFPR05	95.30%	94.10%	95.10%
CarNet (with window size 7)	PUCPR	96.20%	94.20%	96.30%
	UFPR04	96.30%	94.80%	95.10%
	UFPR05	96.30%	95.20%	95.90%
CarNet (with window size 9)	PUCPR	92.70%	92.20%	93.90%
	UFPR04	94.90%	95.30%	93.70%
	UFPR05	93.10%	94.30%	94.20%
CarNet (with window size 11)	PUCPR	**98.80%**	**94.40%**	**97.70%**
	UFPR04	**98.30%**	**95.60%**	**97.60%**
	UFPR05	**98.40%**	**95.20%**	**97.50%**
CarNet (with window size 13)	PUCPR	97.40%	93.30%	95.80%
	UFPR04	97%	95.40%	96.20%
	UFPR05	97.10%	95.10%	96%

**Table 9:** Results related to PKLot [4] dataset. Comparison of training and validation accuracies for CarNet with well-known architectures.

Name of Method	Training Accuracy	Validation Accuracy
AlexNet [6]	**99.98%**	**98.82%**
CarNet	97.23%	90.70%
VGG16 [52]	99.38%	93.72%
VGG16 (fine-tuned) [52]	99.94%	94.44%
VGG19 [52]	99.94%	95.25%
VGG19 (fine-tuned) [52]	99.96%	94.54%
Xception [53]	99.92%	98.53%
Inception V3 [54]	99.61%	97.73%
ResNet50 [55]	99.51%	98.02%

**Table 10:** Results related to PKLot dataset [4]. Comparison of testing accuracies for Carnet with respect to other models.

Name of Method	Name of Training Subset	Testing Accuracy		
		PUCPR	UFPR04	UFPR05
AlexNet [6]	PUCPR	98.60%	88.80%	83.40%
	UFPR04	89.50%	**98.20%**	87.60%
	UFPR05	88.20%	87.30%	**98%**
CarNet	PUCPR	**98.80%**	**94.40%**	**97.70%**
	UFPR04	**98.30%**	95.60%	**97.60%**
	UFPR05	**98.40%**	**95.20%**	97.50%
VGG16 [52]	PUCPR	88.20%	94.20%	90.80%
	UFPR04	89.70%	95.30%	90%
	UFPR05	90.50%	94.90%	91.80%
VGG16 (fine-tuned) [52]	PUCPR	69.10%	91.70%	92.60%
	UFPR04	67.90%	95%	86.80%
	UFPR05	91.20%	92.80%	92.80%
VGG19 [52]	PUCPR	81.50%	93.80%	94.60%
	UFPR04	80.40%	92.30%	91.90%
	UFPR05	88.80%	95.10%	95.90%
VGG19 (fine-tuned) [52]	PUCPR	84.70%	94.10%	91.90%
	UFPR04	85.40%	94.10%	92.30%
	UFPR05	87.60%	94.30%	95.40%
Xception [53]	PUCPR	96.30%	92.50%	93.3
	UFPR04	94%	94.60%	93.40%
	UFPR05	95.70%	90.90%	91.20%
Inception V3 [54]	PUCPR	90.80%	91.10%	94.2
	UFPR04	91.70%	95.20%	92.40%
	UFPR05	94.30%	92.9	93.70%
ResNet50 [55]	PUCPR	90.50%	93.90%	94.10%
	UFPR04	93.70%	94.80%	93.30%
	UFPR05	92.20%	94.80%	95.50%

**Table 11:** Comparisons of training and validation accuracies for CarNet, AlexNet [6] and ResNet50 [55] on CNRPark + EXT dataset [5].

Name of Method	Training Accuracy	Validation Accuracy
CarNet	97.91%	90.05%
AlexNet [6]	**96.99%**	**97.91%**
ResNet50 [55]	96.51%	97.80%

**Table 12:** Comparisons of testing scores for CarNet, AlexNet [6] and ResNet50 [55] on CNRPark + EXT dataset [5].

Name of Method	Testing Accuracy
CarNet	**97.24%**
AlexNet [6]	96.54%
ResNet50 [55]	96.24%

**Table 13:** Comparison of testing results of CarNet and mAlexNet [5] on PKLot dataset [4].

Name of Architecture	Training Subset	Testing Subset	Accuracy (%)	Mean (%)
**mAlexNet [5]**	UFPR04	UFPR04	**99.54**	96.74
	UFPR04	UFPR05	93.29	
	UFPR04	PUCPR	98.27	
	UFPR05	UFPR04	93.69	
	UFPR05	UFPR05	**99.49**	
	UFPR05	PUCPR	92.72	
	PUCPR	UFPR04	**98.03**	
	PUCPR	UFPR05	96	
	PUCPR	PUCPR	**99.9**	
**CarNet**	UFPR04	UFPR04	95.6	**97.04**
	UFPR04	UFPR05	**97.6**	
	UFPR04	PUCPR	**98.3**	
	UFPR05	UFPR04	**95.2**	
	UFPR05	UFPR05	97.5	
	UFPR05	PUCPR	**98.4**	
	PUCPR	UFPR04	94.4	
	PUCPR	UFPR05	**97.6**	
	PUCPR	PUCPR	98.8	

**Table 14:** Comparison of testing results of CarNet and mAlexNet [5] on PKLot [4] and CNRPark + EXT datasets [5].

Name of Architecture	Training Dataset	Testing Dataset	Accuracy (%)	Mean (%)
**mAlexNet [5]**	PKLot [4]	CNRPark + EXT [5]	83.88	88.70
	CNRPark + EXT [5]	PKLot [4]	84.53	
	CNRPark + EXT [5]	CNRPark + EXT [5]	97.71	
**CarNet**	PKLot [4]	CNRPark + EXT [5]	**94.77**	**97.03**
	CNRPark + EXT [5]	PKLot [4]	**98.21**	
	CNRPark + EXT [5]	CNRPark + EXT [5]	**98.11**	

**Table 15:** Comparison of AUC scores of CarNet with Martin et al. (LR) [47] and Martin et al. (SVM) [47] on PKLot dataset.

Name of Architecture	Training Set	Testing Test	AUC Score	Method Achieved Best Result
**CarNet**	UFPR04	UFPR04	97.9	PKLot [4]
**Martin et al. (LR) [47]**	UFPR04	UFPR04	0.9994	
**Martin et al. (SVM) [47]**	UFPR04	UFPR04	0.9996	
**PKLot [4]**	UFPR04	UFPR04	**0.9999**	
**CarNet**	UFPR04	UFPR05	**99.35**	CarNet
**Martin et al. (LR) [47]**	UFPR04	UFPR05	0.9928	
**Martin et al. (SVM) [47]**	UFPR04	UFPR05	0.9772	
**PKLot [4]**	UFPR04	UFPR05	0.9595	
**CarNet**	UFPR04	PUCPR	**99.82**	CarNet
**Martin et al. (LR) [47]**	UFPR04	PUCPR	0.9881	
**Martin et al. (SVM) [47]**	UFPR04	PUCPR	0.9569	
**PKLot [4]**	UFPR04	PUCPR	0.9713	
**CarNet**	UFPR05	UFPR04	97.96	Martin et al. (LR) [47]
**Martin et al. (LR) [47]**	UFPR05	UFPR04	**0.9963**	
**Martin et al. (SVM) [47]**	UFPR05	UFPR04	0.9943	
**PKLot [4]**	UFPR05	UFPR04	0.9533	
**CarNet**	UFPR05	UFPR05	99.89	PKLot [4]
**Martin et al. (LR) [47]**	UFPR05	UFPR05	0.9987	
**Martin et al. (SVM) [47]**	UFPR05	UFPR05	0.9988	
**PKLot [4]**	UFPR05	UFPR05	**0.9995**	
**CarNet**	UFPR05	PUCPR	**97.91**	CarNet
**Martin et al. (LR) [47]**	UFPR05	PUCPR	0.9779	
**Martin et al. (SVM) [47]**	UFPR05	PUCPR	0.9405	
**PKLot [4]**	UFPR05	PUCPR	0.9761	
**CarNet**	PUCPR	UFPR04	**98.45**	CarNet
**Martin et al. (LR) [47]**	PUCPR	UFPR04	0.9829	
**Martin et al. (SVM) [47]**	PUCPR	UFPR04	0.9843	
**PKLot [4]**	PUCPR	UFPR04	0.9589	
**CarNet**	PUCPR	UFPR05	**99.38**	CarNet
**Martin et al. (LR) [47]**	PUCPR	UFPR05	0.9457	
**Martin et al. (SVM) [47]**	PUCPR	UFPR05	0.9401	
**PKLot [4]**	PUCPR	UFPR05	0.9152	
**CarNet**	PUCPR	UFPR05	99.86	PKLot [4]
**Martin et al. (LR) [47]**	PUCPR	UFPR05	0.9994	
**Martin et al. (SVM) [47]**	PUCPR	UFPR05	0.9994	
**PKLot [4]**	PUCPR	UFPR05	**0.9999**	
